# An Inductorless Gain-Controllable Wideband LNA Based on CCCIIs

**DOI:** 10.3390/mi13111832

**Published:** 2022-10-26

**Authors:** Qiuzhen Wan, Jiong Liu, Simiao Chen

**Affiliations:** College of Information Science and Engineering, Hunan Normal University, Changsha 410081, China

**Keywords:** low noise amplifier (LNA), wideband, inductorless, gain-controllable, current conveyor, impedance matching circuit

## Abstract

In this paper, an inductorless and gain-controllable 0.5~2.5 GHz wideband low noise amplifier (LNA) based on second generation current controlled current conveyors (CCCIIs) is presented. The proposed wideband LNA utilizes CCCIIs as building blocks to implement the amplifier stage and impedance matching stage. By varying the DC biasing current of the CCCII, the voltage gain of the proposed LNA is controllable in the range of 1~18 dB. In the frequency range of 0.5~2.5 GHz, the post-layout simulation results show that the proposed LNA has a typical voltage gain S21 of 12.6 dB with a gain ripple of ±1.5 dB, an input and output return loss (S11 and S22) of, respectively, −21.4 dB to −16.6 dB and −18.6 dB to −10.6 dB, and a high reverse isolation S12 of −65.2 dB to −39.5 dB. A noise figure of 4.05~4.35 dB is obtained across the whole band, and the input third-order intercept point (IIP3) is −2.5 dBm at 1.5 GHz. Using a 0.18 μm RF CMOS technology, the LNA occupies an active chip area of only 0.096 mm^2^ with a power consumption of 12.0 mW.

## 1. Introduction

In the last few years, in the software-defined and multi-standard wireless communication systems, a radio frequency (RF) transceiver must be able to support several applications simultaneously, such as 3G/4G/5G mobile communication network, wireless local area network (WLAN), wireless sensor network (WSN), ZigBee (IEEE 802.15.4), and many others [1,2,3,4]. Therefore, the research of the wideband RF transceiver front-end is significant and requires the research of RF circuits, whose impedances are matched over wide bands and whose frequency characteristics are stable over several GHz.

As the first active block after the antenna, the wideband LNA constitutes one of the essential elements in a wideband RF front-end [5,6]. The most important parameters of wideband LNA are voltage gain, noise figure (NF), bandwidth, linearity, impedance matching, power consumption, and chip area. The goals in wideband LNA design include providing moderate to high gain with good linearity, minimizing its noise figure to enhance the sensitivity of a receiver, and establishing wideband input/output impedance matching to reduce the return loss. The additional constraints of low power consumption and small chip area are imposed in portable systems.

In the published literature, various wideband LNA circuit techniques in submicron technologies have been proposed, such as a filter matching network amplifier, distributed amplifier, feed-forward noise canceling amplifier, and resistive shunt feedback amplifier. The filter matching network amplifier can consume a small amount of power and achieve wideband input impedance matching. However, the utilization of the filter at the input needs a great deal of inductor components [7,8,9]. The distributed amplifier is usually used to improve voltage gain at high frequency and therefore can extend the bandwidth, which has the inherent advantages of temperature-insensitive wideband input/output impedance matching and wideband voltage gain, but it dissipates a large chip area owing to the cascade of several stages [10,11]. The feed-forward noise canceling amplifier has been proposed to break the trade-off between input impedance matching and noise figure. However, its bandwidth is limited without the use of the inductor components [12,13]. The resistive shunt feedback amplifier can improve gain flatness and provide wideband input impedance matching, but it is difficult to satisfy noise figure and voltage gain requirements simultaneously [14,15].

As can be seen from the above techniques, in the design of wideband LNAs, the inductor components are extensively used to design impedance matching circuits, resonator networks and to widen the −3 dB bandwidth. However, the on-chip inductor components are large in size and cannot be converted easily from one technology to the next. The on-chip inductor components can result in a significant increase in the chip area and therefore the implementation cost. On the other hand, the feasibility of a wideband LNA to multi-mode and multi-standard is not only dependent on its bandwidth, it should also be able to support variable signal gain according to necessity. For example, when the linearity requirement is more important, a moderate gain is required because the linearity is usually inversely proportional to the gain. When the receiver’s NF is required for good performance, the LNA needs to provide high gain to decrease noise contributions of the latter stages. The wideband LNA should be gain-controllable so that it can moderate gain amplification of the strong signals and high gain amplification of the weak signals.

In this paper, a new design for the inductorless gain-controllable wideband LNA using second generation current controlled current conveyors (CCCIIs) has been presented and simulated in a 0.18 μm RF CMOS technology. For the realization of this LNA, three different CCCIIs, as the main building blocks, which are symbolized CCCII 1 to CCCII 3, can provide voltage signal amplification and wideband output impedance matching. Compared to other wideband LNA designs, this kind of LNA can exhibit a smaller chip area, a wider gain-controllable range, and a good high frequency performance.

This paper is organized as follows. In Section 2, the operation principle and some RF applications of current conveyor are discussed, then, the design schematic of a new LNA based on CCCIIs is analyzed. In Section 3, the post-layout simulation results of the proposed wideband LNA are introduced, meanwhile, we present comparisons with the recent works to show the advantages of the new LNA. Finally, a conclusion on this LNA is given in Section 4.

## 2. Operational Principle and Circuit Implementation

### 2.1. Current Conveyor Principle of Operation

The current conveyors (CCs) can be used with the other active/passive components in a specific circuit configuration to implement many analogue signal functions [16,17]. Usually, CCs simplify analogue circuit design to implement functions, such as impedance converters, filters, gyrators, inductances, oscillators, and operational amplifiers, etc. In the last few years, CCs have also received much attention for their use as RF basic building blocks [18,19]. The advantages of using CCs in RF circuit design are that they do not require inductor components to make the impedance matching circuit and that the voltage gain can be made controllable by only varying a DC biasing current.

**Figure 1:**
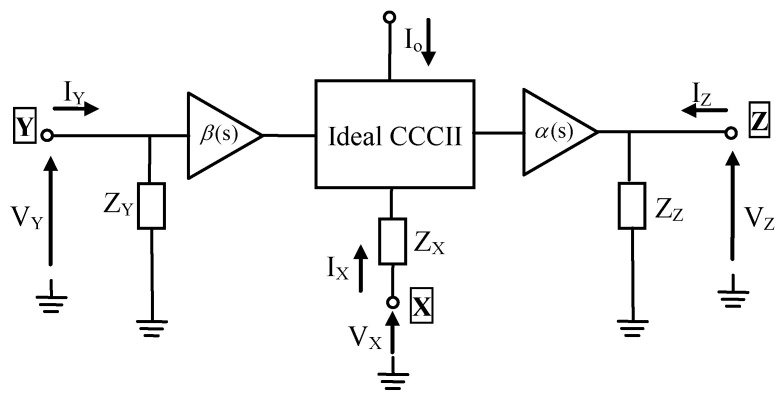
Equivalent circuit of CCCII.

In this paper, the use of second-generation current conveyor (CCII) to implement the wideband RF LNA has been proposed. The CCII is an active device comprised of the three ports, denoted port X, port Y, and port Z. Far from being ideal in their operation, the three ports of the CCII are beset by many parasitic elements, represented by the impedances Z_X_ (R_X_ in series with L_X_), Z_Y_ (R_Y_ in series with C_Y_), and Z_Z_ (R_Z_ in series with C_Z_) at ports X, Y, and Z, respectively. Here, the Z_Y_ and Z_Z_ are impedances with very high values. While α(s) and β(s) represent the unity current (from port X to port Z) and voltage (from port Y to port X) transfer functions, ideally α(s) and β(s) are equal to 1. As shown in Figure 1, the value of these impedances, in particular Z_X_ (with a very small value), can be changed by varying the DC biasing current I_o_ of the conveyor, giving rise to the concept of second generation current controlled current conveyor (CCCII).

The governing equation of the CCCII can be given in matrix form as:

(1) [IYVXIZ]=[1ZY(Io)00β(s)ZX(Io)00α(s)1ZZ(Io)]⋅[VYIXVZ]

The CCCII acts as a current follower (between port X and port Z), a voltage follower (between port Y and port X), and a transconductor (between port Z and port Y). The possibility of controlling Z_X_ (mainly consists of the parasitic resistance R_X_) using the DC biasing current I_o_, has made it to extend the applications of CCCIIs to the domain of controlled electronic functions.

### 2.2. The CCCII-Based LNA Principle of Operation

**Figure 2:**
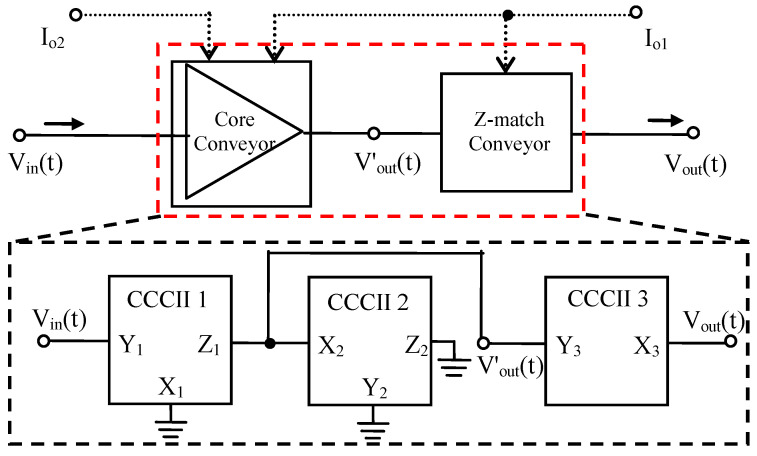
The operational principle of CCCII-based LNA.

The CCCII is an increasingly popular building block for RF functions [20,21,22]. The CCCII can be used to design a gain-controllable LNA, the gain of which can be controlled by the DC biasing current I_o_. For the realization of this LNA, three different CCCIIs, as the main building blocks, which are symbolized as CCCII 1 to CCCII 3, can provide voltage signal amplification and wideband output impedance matching when connected, as shown in Figure 2. The current conveyor CCCII 1 converts the input voltage signal into a current signal, the connection of the two current conveyors, CCCII 1 and CCCII 2, amplify this current signal, and the current conveyor CCCII 2 converts the amplified current signal into the output voltage signal. Besides, the current conveyor CCCII 3 is the Z-match block on the output of the LNA, which adjusts the output impedance of the LNA to an ideal value.

The input voltage signal V_in_(t) is provided at port Y_1_ of the current conveyor CCCII 1. Port Z_1_ of current conveyor CCCII 1 and port X_2_ of the current conveyor CCCII 2 are connected to each other, and the output voltage signal $V_{\mathrm{out}}^{\prime }$(t) is tapped at this common connection. Assuming Z_X1_ and Z_X2_ to be purely resistive in nature (as L_X1_ and L_X2_ are the negligible parasitic inductances), with R_X1_ and R_X2_, V_in_(t) is first converted by R_X1_ into I_X1_(t), which is given by

(2) $$I_{X1}(t)=-\frac{V_{in}(t)}{R_{X1}}$$

I_X1_(t) is copied to port Z_1_ as I_Z1_(t) (as to the current conveyor CCCII 1 between its port X_1_ and port Z_1_ is a current follower). Because of the connection of the two current conveyors CCCII 1 and CCCII 2, this current signal I_Z1_ comes into port X_2_ of the current conveyor CCCII 2. Therefore, the current signal I_X1_ = I_Z1_ = I_X2_ can be converted to a voltage signal owing to the parasitic resistance R_X2_ of CCCII 2. The resulting output voltage signal $V_{\mathrm{out}}^{\prime }$(t) is given by

(3) $$V_{out}^{\prime }(t)=-R_{X2}\cdot I_{X1}(t)=\frac{R_{X2}}{R_{X1}}\cdot V_{in}(t)$$

In a typical CCCII operation, the parasitic resistance R_X_ at port X is inversely proportional to the DC biasing current I_o_ of the current conveyor, which is the fundamental property of the CCCII [20]. Then, the voltage gain (G) of the designed LNA is given by

(4) $$G=\frac{V_{out}^{\prime }(t)}{V_{in}(t)}=\frac{R_{X2}}{R_{X1}}=\frac{I_{o1}}{I_{o2}}$$

where R_x1_ and R_x2_ represent the parasitic resistances at port X of the corresponding current conveyor and I_o1_ and I_o2_ are their respective DC biasing currents. Under a certain DC biasing current I_o2_ range condition, the inverse relationship of voltage gain G to DC biasing current I_o2_ enables higher voltage gain at lower DC biasing current and, thus, at lower power consumption.

### 2.3. The CCCII-Based LNA Circuit Design

**Figure 3:**
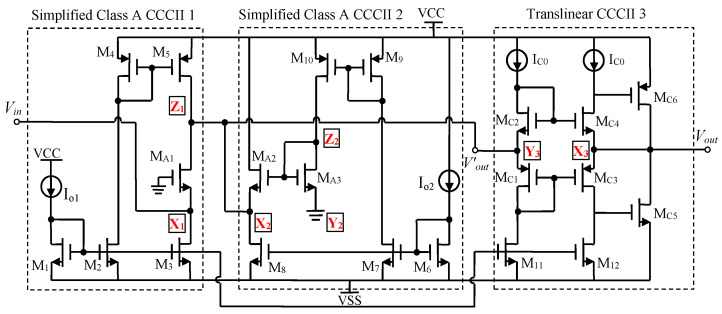
The schematic of the proposed CCCII-based LNA.

One of the base demands in the wideband RF LNA design is to have 50 Ohm input impedance matching [5]. According to the schematic as shown in Figure 2, the input signal is fed at port Y_1_ of CCCII 1, at which intrinsic impedance Z_Y1_ (R_Y1_ in series with C_Y_) at port Y_1_ is of some tens or hundreds of kOhm. However, port X_1_ of CCCII 1 has impedance R_X1_ lower than some hundreds of Ohm. On the other hand, the impedance R_X1_ can be reduced to 50 Ohm for higher values of the DC biasing current I_o1_. Hence, the input signal is changed from port Y_1_ to port X_1_. The following optimization of the circuit architecture leads to the schematic of the proposed CCCII-based LNA as presented in Figure 3.

As can be seen from Figure 3, the proposed LNA utilizes two simplified class A CCCIIs [23] as building blocks to implement the amplifier stage. The current conveyor CCCII 1 consists of an input common gate gain stage M_A1_ and the other current mirror transistors M_1_~M_5_, and the current conveyor CCCII 2 consists of a source follower stage M_A2_–M_A3_ and the other current mirror transistors M_6_~M_10_. The output of the input transistor M_A1_ is connected to the input of the source follower M_A2_. Note that the core of the proposed LNA comprises only three NMOS transistors (M_A1_~M_A3_) in its signal path, thereby allowing for a higher bandwidth to be obtained and improving the noise performance. The current sources I_o1_ and I_o2_ are used to bias the main NMOS transistors M_A1_ and M_A2_–M_A3_, as explained later, to adjust the voltage gain and to match the input impedance without the inductor components.

Based on a first order analysis in the wideband operation mode of Figure 3, a voltage gain can be given by

(5) $$G(s)=\frac{V_{out}^{\prime }(s)}{V_{in}(s)}=\frac{g_{m1}}{g_{m2}+C_{T}s}$$

where C_T_ is the total parasitic capacitance at the output node of port Z_1_ with the input node of port X_2_ and port Y_3_, while g_m1_ and g_m2_ are the transconductances of M_A1_ and M_A2_, respectively. This transfer function obtains a single dominant pole frequency, which approximately determines the amplifier bandwidth by

(6) $$f_{-3dB}=\frac{g_{m2}}{2\pi C_{T}}$$

And the DC voltage gain approximately by

(7) $$G=\frac{g_{m1}}{g_{m2}}$$

At the low frequency band, the input impedance can be approximately described by

(8) $$Z_{in}=\frac{1}{g_{m1}}$$

Equations (6) and (7) show that a compromise on g_m1_ and g_m2_, hence on the transistor sizes, as well as the DC biasing currents I_o1_ and I_o2_ of M_A1_ and M_A2_, respectively, is necessary to optimize voltage gain and bandwidth. As can be seen, I_o1_ through g_m1_ affects the gain but has a little effect on the bandwidth, whereas I_o2_ controls both gain and bandwidth through g_m2_. Therefore, varying I_o2_, g_m2_ can be varied and the gain bandwidth product can be suitably adjusted. Increasing I_o1_ boosts the gain up again, but should be optimized to keep the power consumption sufficiently low. Under a certain DC biasing current I_o2_ range condition, the inverse relationship of voltage gain G to DC biasing current I_o2_ enables higher gain at lower DC biasing current and, therefore, at lower power consumption. Meanwhile, without the need of an LC matching network, Equation (8) indicates Z_in_ can be matched to the source impedance (50 Ohm) through I_o1_. From Figure 3, the proposed wideband LNA is an inductorless circuit, its chip area is smaller than the most other LNAs.

In Figure 3, the number of components in the signal chain is low: only three NMOS transistors M_A1_~M_A3_. The main noise sources of the proposed LNA are contributed by the input NMOS transistor M_A1_. When neglecting the parasitic capacitances and the noise of I_o1_ and I_o2_, the noise figure of the LNA is approximately given by

(9) $$NF=1+\gamma (1+\frac{g_{m2}}{g_{m1}}+\frac{g_{m5}}{g_{m1}})$$

The excess noise factor γ is a constant that depends on the transistor size, and g_m5_ represents the transconductance of M_5_. As shown from Equation (9), the noise figure decreases when g_m1_ is increased. It can be achieved by increasing the transistor size of M_A1_ or the DC biasing current I_o1_.

### 2.4. The Output Impedance Matching Design

In Figure 2, the Z-match block on the output is the impedance conversion circuit, which uses a translinear CCCII in the voltage follower mode to adjust the output impedance of the LNA to an ideal value. This design makes the output impedance of the LNA fully independent of its gain. In Figure 3, the core of translinear CCCII is composed of NMOS transistors (M_C2_ and M_C4_) and PMOS transistors (M_C1_ and M_C3_) as a translinear loop. At the same time, the transistors M_C6_ and M_C4_, respectively, M_C5_ and M_C3_, implement the other feedback loops to adjust the output impedance at port X_3_ of the conveyor CCCII 3. The DC biasing current I_o1_ is copied to the required branches using current mirror transistors M_1_ and M_11_–M_12_. The signal to be adapted, $V_{\mathrm{out}}^{\prime }$, is fed at port Y_3_ of the current conveyor CCCII 3 ($V_{\mathrm{out}}^{\prime }$ = V_Y3_), which can be considered an open circuit since its intrinsic resistance, R_Y3_, is very high. The output is tapped at port X_3_ of the current conveyor CCCII 3 (V_out_ = V_X3_). The voltage transfer function β(s) (ideally equal to 1) of the voltage follower mode is

(10) $$\beta (s)=\frac{V_{X3}(s)}{V_{Y3}(s)}=\frac{V_{out}(s)}{V_{out}^{\prime }(s)}\approx 1$$

The output impedance, at port X_3_ of the conveyor CCCII 3, consists of resistance R_X3_ in series with a negligible parasitic inductance L_X3_. By changing the DC biasing current I_o1_ of the conveyor, different resistance R_X3_ values can be obtained.

As can be seen in Figure 3, the CCCII-based output impedance matching circuit in this section has several advantages. Firstly, according to the Friis equation [24], the proposed impedance matching circuit is located at the output of the LNA, so its insertion has a little effect on the overall noise figure. Secondly, the gain-controllable characteristic of the LNA is not impacted by the insertion of the matching circuit, therefore there is no attenuation of the amplifying signal. Thirdly, the additional transistors in the signal path led to an increased immunity to reverse signal propagation. As a result, the insertion of the matching circuit led to the output impedance being perfectly matched to the ideal value (50 Ohm).

Compared to the traditional passive component solutions, the output impedance matching circuit of the proposed CCCII-based LNA is vastly superior by being: the small chip area without the inductor components, the flexible matching circuit to adjust any impedance to the ideal value, and a novel example of wideband output matching for the RF LNA applications.

## 3. Simulation Results and Discussion

**Figure 4:**
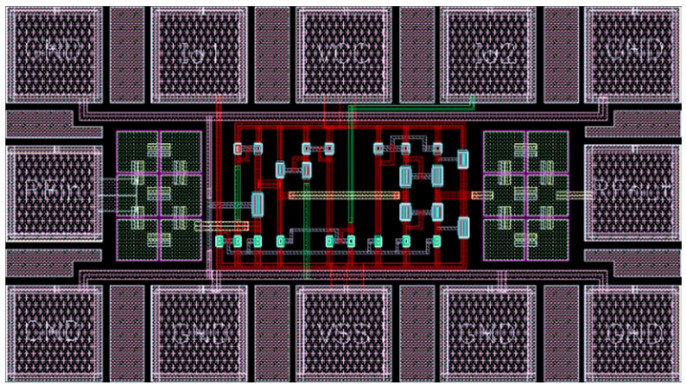
The layout diagram of the proposed wideband LNA.

The proposed wideband LNA has been realized using a GlobalFoundries’ 0.18 μm single-poly six-metal CMOS technology and simulated using Cadence SpectreRF simulator. The GlobalFoundries’ Design Rule (YI-093-DR001_Rev1V_1.8V-3.3V) and GlobalFoundries’ Spice Model spec (yi093dr001_1v_00_20090731a) are used. During the design process, a very important step is the realization of the layout. To maximize RF performance, the layout was made by following classical design rules commonly used for analogue RF circuit synthesis (e.g., transistor matching, symmetric design, etc.). In order to validate the design approach, optimization of biasing currents and width of transistors with extensive DC analysis are simulated. The layout of the proposed wideband LNA is presented in Figure 4, which takes an active chip area, including testing pads of 0.42 mm × 0.23 mm. The absence of inductor components results in a very small chip area (0.096 mm^2^).

**Figure 5:**
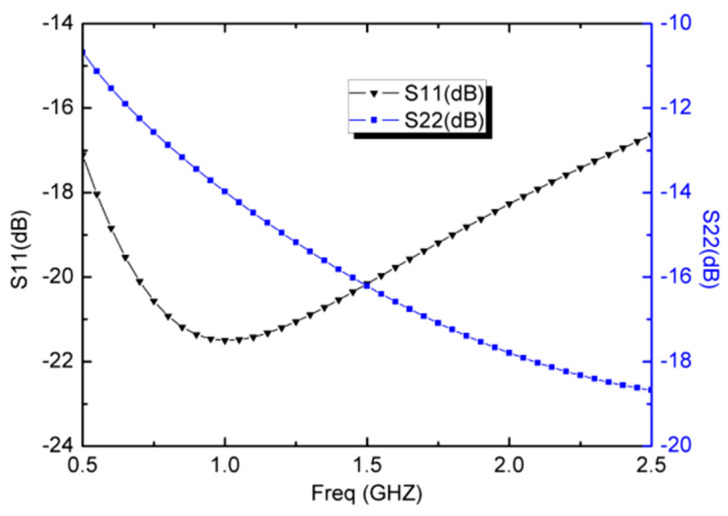
Simulated S11 and S22 of the wideband LNA.

Figure 5 presents the simulated input return loss (S11) with I_o1_ = 1.25 mA, I_o2_ = 50 μA, and V_DC_ = ±1.5 V. At a frequency of 1.0 GHz, S11 has a value of −21.4 dB and it remains below −16.6 dB for frequencies from 0.5 GHz to 2.5 GHz. As a critical parameter of the LNA, the S11 provides a good wideband input matching performance without the LC matching network. Figure 5 also presents the simulated output return loss (S22), which uses the translinear CCCII 3 in voltage follower mode as the output impedance matching circuit. The simulated S22 is from −10.6 dB to −18.6 dB over the entire frequency band of 0.5~2.5 GHz, indicating a reasonably acceptable output matching condition.

**Figure 6:**
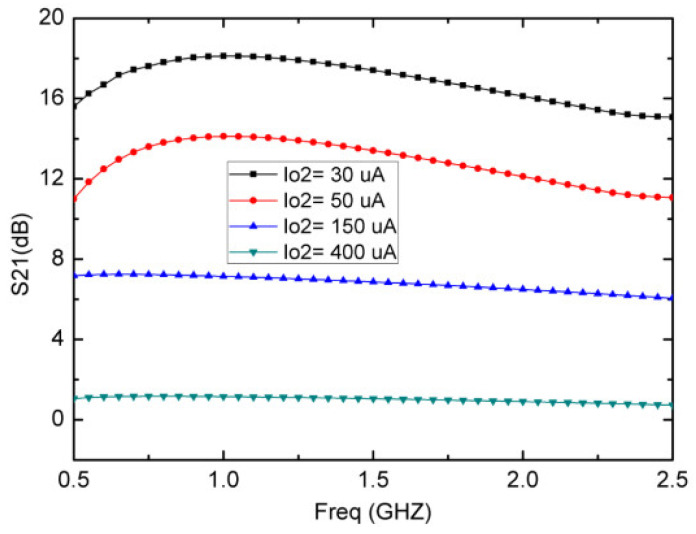
Simulated gain controllable range for various I_o2_ with I_o1_ = 1.25 mA and V_DC_ = ±1.5 V.

In the proposed LNA, gain control over a wide range is achieved by varying the DC biasing current, I_o2_, of the CCCII 2. Figure 6 shows that the voltage gain of the proposed LNA can be controllable between 18 dB and 1 dB when I_o2_ varies between 30 μA and 400 μA, at the same time, the LNA consumes between 3.8 mA and 5 mA for the entire range of I_o2_. The inversely proportional relationship between the voltage gain and the DC biasing current indicates that better gain is achieved at the lower power consumption.

**Figure 7:**
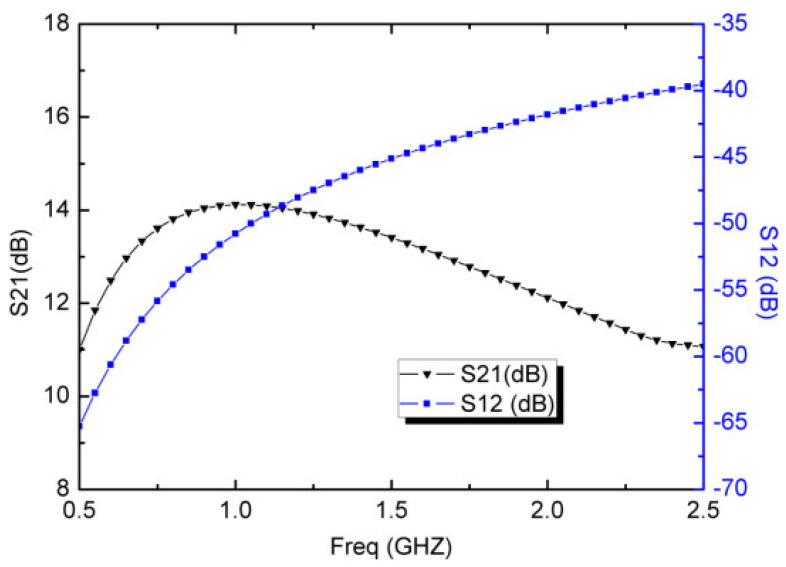
Simulated S21 and S12 of the wideband LNA.

As described by Equations (6) and (7), the voltage gain increased and bandwidth decreased when I_o2_ decreased. In order to control the LNA performance over the 0.5~2.5 GHz bandwidth of interest, the currents I_o1_ and I_o2_ are fixed at 1.25 mA and 50 μA for this design. Under these conditions, the typical voltage gain (S21) and reverse isolation (S12) performances are present in Figure 7. The simulated S21 shows a typical voltage gain of 12.6 dB with a gain ripple of ±1.5 dB from 0.5 GHz to 2.5 GHz. According to Figure 7, the simulated S12 is from −65.2 dB to −39.5 dB in the required band, which indicates that the designed LNA can achieve a good stability characteristic. The better S12 can decrease the subsequent local oscillation (LO) leakage, arising from the substrate coupling and the capacitive paths.

**Figure 8:**
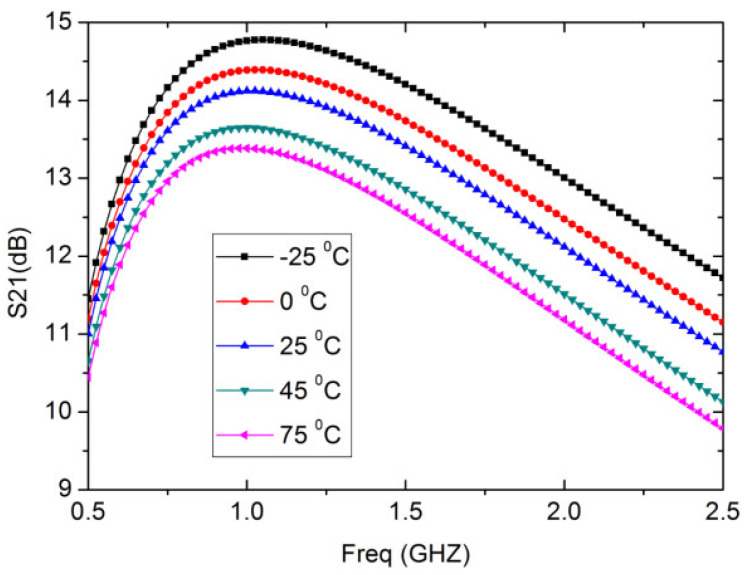
Simulated S21 of the wideband LNA with temperature variations.

As shown in Figure 8, the proposed wideband LNA is simulated for different temperatures and corner processes. The wideband LNA designed here has an excellent thermal stability: in the temperature range of −25 °C to 75 °C, the gain S21 drops about by ±1 dB. As the temperature increases, the gain S21 of the wideband LNA gradually decreases.

**Figure 9:**
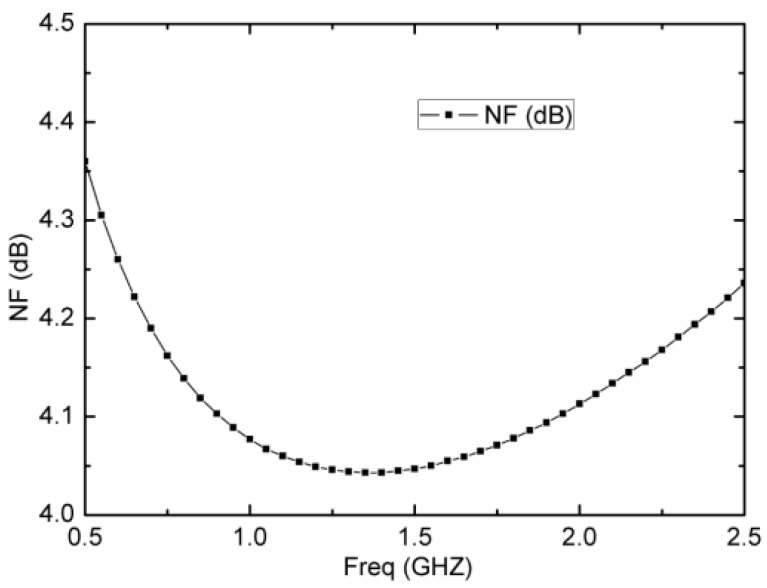
Simulated noise figure of the wideband LNA.

Owing to noise concerns, Figure 9 shows the simulated NF of the wideband LNA with I_o1_ = 1.25 mA and I_o2_ = 50 μA. It has a relatively flat and low NF performance across the entire 2 GHz bandwidth. From Figure 9, an excellent NF of 4.05~4.35 dB in the frequency range of 0.5~2.5 GHz is achieved. The maximum NF is 4.35 dB at 0.5 GHz and the minimum NF is 4.05 dB at 1.5 GHz, with an average NF of 4.2 dB.

**Figure 10:**
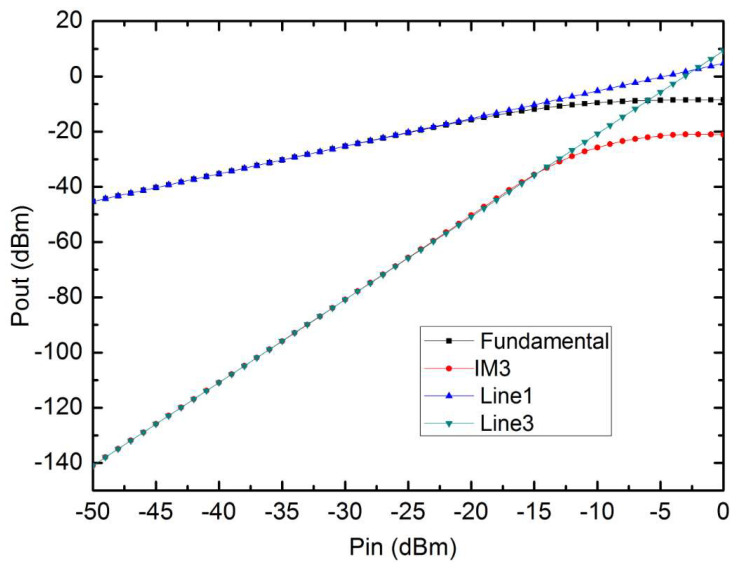
Simulated IIP3 and P1dB of the wideband LNA.

To observe the nonlinear behavior, the two-tone signals at 1.50 GHz and 1.51 GHz with equal power levels are applied to the wideband LNA. The two-tone signals with 10 MHz spacing are used to simulate the fundamental and third-order inter-modulation (IM3) output power, versus input power, characteristics. As shown in Figure 10, an input third-order intercept point (IIP3) of −2.5 dBm and an input 1 dB compression point of −12.5 dBm are achieved. These results show that the designed LNA can achieve good linearity even with the low NF.

For I_o2_ = 50 μA, and at a dual supply voltage of ±1.5 V, the CCCII-based LNA consumes a current of 4 mA which results in a total power consumption of 12 mW. Finally, Table 1 is a summary of the proposed CMOS 0.5~2.5 GHz wideband LNA and reports recent CMOS state-of-the-art wideband LNA designs. Compared to other wideband LNA designs, the post-layout simulation results show that the proposed LNA can achieve a good wideband input/output impedance matching by using the CCCIIs, and further benefits include its inductorless design with smaller chip area and the capability of gain-controllability by varying a DC biasing current (I_o2_).

## 4. Conclusions

In this paper, a new wideband LNA which utilizes current conveyors as building blocks has been presented. Thanks to the use of three different CCCIIs for conveying the signal, the proposed wideband LNA has the following notable advantages over recent works: the total absence of inductor components, and thus, the LNA has an active chip area of only 0.096 mm^2^; wideband input/output impedance matching and stable frequency responses in the required 0.5~2.5 GHz band; easily gain-controllable over a wide range (from 1 dB to 18 dB). The above excellent performances have shown that the proposed CCCII-based wideband LNA is suited to the software-defined and multi-standard wireless communication systems.

## Figures and Tables

**Table 1:** Performance summary of the proposed LNA and comparison with other designs.

Specification	[6] ^S^	[7] ^S^	[8] ^M^	[13] ^S^	[14] ^M^	This Work ^S^
**Technology**	65 nm CMOS	0.18 μm CMOS	0.13 μm CMOS	0.18 μm CMOS	0.18 μm CMOS	0.18 μm CMOS
**Frequency (GHz)**	0.5~5	2.3~4.8	3.1~4.8	2~3.3	3.7~11.9	0.5~2.5
**Input return loss S11 (dB)**	<−10	<−10	<−8.4	<−10	<−8.5	<−16.6
**Output return loss S22 (dB)**	<−10	--	<−14	--	<−10	<−10.6
**Voltage gain maximum (dB)**	13	24	13	16.8	8.1	14.1
**Reverse isolation S12 (dB)**	--	--	<−40	--	<−30.3	<−39.5
**NF (dB)**	4~4.5	2.8–3.7	3.5~4.5	5.55~6	3.02~3.86	4.05~4.35
**IIP3 (dBm)**	−10	−3.2	−6.1	0.5	−1.3	−2.5
**Power dissipation (mW)**	5.0 ^#^	13.1	3.4	2.55 ^#^	7.3	12.0
**Die size (mm^2^)**	0.173	0.34	0.40	0.283	0.34	0.096

^#^ Without the output buffer dissipation. **^S^** The post-layout simulation result. **^M^** The measurement result.

## Data Availability

The data that support the findings of this study are available within the article.
